# 肺癌合并COPD手术及术后复发风险的影响值得关注的几个问题

**DOI:** 10.3779/j.issn.1009-3419.2018.03.22

**Published:** 2018-03-20

**Authors:** 如文 王

**Affiliations:** 陆军军医大学大坪医院全军胸外科研究所 Institute of Thoracic Surgery, Daping Hospital, Army Military Medical University

肺癌与慢性阻塞性肺疾病（chronic obstructive pulmonary disease, COPD）同是呼吸系统常见病、多发病，除其存在发病率高、死亡风险大的临床特点外，且两者还存在一些相同的发病因素，如高龄、吸烟、男性等，更为重要的是罹患COPD的人群发生肺癌比例显著高于非COPD人群，这提示可能COPD与肺癌的发生、发展以及预后有着密切的相关性和内在联系，同时COPD的存在对肺癌病人围手术期处理、手术方式的选择及对远期疗效的影响等问题均值得关注。

## 1 肺癌合并COPD患者加速康复外科对肺功能改善的作用

各脏器功能状态是评价病人能否耐受手术及影响术后康复的重要指标，尤其是肺功能对胸外科手术甚为重要。肺癌合并COPD患者除存在较大手术风险外，对术后恢复亦有很大困难。目前加速康复外科理念指导下应用肺康复训练方法，对改善肺功能具有积极的作用，这不仅能显著降低手术风险，还可明显减少术后并发症，据研究肺功能的改善还能使患者长期生存获益。杨鲁民等^[1]^对26例中重度COPD合并肺癌患者行手术切除。围手术期给予支气管扩张剂吸入、氧疗、辅助排痰、呼吸康复训练及机械通气等综合治疗。比较术前综合治疗后与术后1个月和3个月时患者肺功能与血气分析情况。26例患者术前经综合治疗后，每分钟最大通气量（maximal voluntary ventilation, MVV）、一秒用力呼气容积（forced expiratory volume in one second, FEV_1_）和用力肺活量（forced vital capacity,  FVC）均明显改善，动脉血氧分压（partial pressure of oxygen, PaO_2_）、血氧饱和度（pulse oxygen saturation, SpO_2_）上升，PaCO_2_显著下降（*P* < 0.01）。术后3个月时各项指标均已达术前水平（*P* > 0.05）。故中重度COPD合并肺癌患者围手术期给予适当的综合治疗能有效改善肺功能，增加手术耐受性，有利于术后肺功能的康复。赖玉田等^[2]^采用前瞻性随机对照临床研究发现，术前短期（1周）综合肺康复训练。实验组采用以雾化吸入普米克令舒、博利康尼；静脉滴注沐舒坦为主的药物康复以及呼吸训练加耐力训练（Nustep）的物理康复；而对照组患者仅按常规术前准进行。结果证实：实验组患者最大峰值流速、6分钟步行距离、能量消耗均有提高；术后住院时间、术后抗生素使用时间均低于对照组。术前短期综合肺康复训练能够提高肺癌合并轻中度COPD患者心肺耐力，加速患者术后快速康复。

## 2 肺癌合并COPD患者手术方式的选择

肺癌合并COPD患者手术方式的选择是又一个需慎重考虑和精准选择的难题。除考虑肺癌本身如何选择手术方式和切除范围外，还应考虑存在低肺功能状态对手术方式和围手术期带来的叠加影响。亚肺叶切除曾作为对肺叶切除耐受力差的肺癌患者的一种妥协性术式而得到推广。近年来，对于肺癌早期（直径≤2 cm的磨玻璃结节），不少研究认为，亚肺叶切除可获得与肺叶切除相当的疗效，这对于合并COPD、手术耐受力差的肺癌患者无疑是可供选择方式。对于肺癌合并COPD患者亚肺叶切除似乎既可保留更多的肺功能，又可让患者获得外科手术确切的治疗效果，还能起到部分肺减容对COPD的双重治疗效果，但亚肺叶切除仍存在局部复发的风险。因此对肺癌合并COPD的患者可参考以下几点选择手术方式：①肺病变≤2.0 cm，非跨肺段生长，且符合亚肺叶切除标准；COPD为均质型，肺功能为中度损害者，可选择肺段或楔形切除的亚肺叶切除，肺功能损害偏重者，以选择肺楔形切除为宜。②肺病变或肿瘤跨肺段生长，肺功能轻度损害者可选择肺叶切除，若肺功能为中度损害同时病变所在肺叶有肺大泡占据了较多的肺功能，亦可考虑肺叶切除。③病变位于肺周围，肺功能损害较重，同时达到肺减容指征者，可行含病变在内的肺减容术。

手术方式的不同，淋巴结清扫范围可异。Ajmani等^[3]^比较了早期肺癌患者接受不同质量肺楔形切除与立体定向放疗相比预后有何差异，发现高质量的楔形切除（切缘阴性，淋巴结采样＞5枚）的患者死亡风险显著降低，并具有显著的生存优势。Hishida等^[4]^在2016年完成的一项回顾性研究发现，基于肺叶的选择性淋巴结清扫（lobe-specific dissection, LSD）5年生存率为81.5%，而系统性淋巴结清扫（systematic node dissection, ND）的5年生存率为75.9%，差异有统计学意义。系统性淋巴结清扫和选择性淋巴结清扫的争议仍然存在，需要更多高质量的研究来进一步探讨。对于肺癌合并COPD患者若选择亚肺叶切除需保证足够的切缘，淋巴结采样数目应大于5枚，以达到高质量局部肺切除标准。若行肺叶切除，对耐受性较差的患者，可行选择性淋巴结清扫；耐受性较好的患者行系统性淋巴结清扫，以获得更为准确的术后病理分期。

## 3 远期疗效的影响

目前对肺癌合并COPD术后复发风险有何影响尚未引起足够的关注，文献亦鲜有报道。本刊报道的《慢性阻塞性肺疾病对非小细胞肺癌术后复发风险的影响》一文，作者回顾性分析了中日友好医院胸外科2012年1月-2015年6月，经胸腔镜行肺叶切除、系统性纵隔淋巴结清扫的421例NSCLC患者的临床资料，比较了249例不伴COPD肺癌患者与172例伴COPD者5年无肿瘤复发生存率，分层分析了COPD严重程度对肿瘤复发的影响，并分析了其他因素对预后影响。结果揭示：合并COPD的NSCLC患者5年无复发生存率为64.0%，显著低于不伴COPD者的78.1%，具有统计学意义（*P*=0.002）。无COPD组、轻度COPD组的5年无复发生存率分别为78.1%和70.4%，差异无统计学意义（*P*=0.115），中重度COPD组5年无复发生存率仅为46.4%，与无COPD组或轻度COPD组之间差异均有统计学意义（*P* < 0.001, *P*=0.001）。随着COPD严重程度增加，术后复发率的风险显著增加。多因素分析结果显示中重度COPD是影响患者预后的独立危险因素，COPD严重程度高危者预后差。

刘德若教授研究团队从COPD罹患NSCLC患者术后远期生存的影响因素入手，探讨了COPD与肺癌术后肿瘤复发之间的关系，得出了较为可靠的结论，具有重要的临床意义，为进一步从慢性炎症出发探讨炎症通路和免疫状态对肺癌的影响，探索发病机制、寻找新的治疗靶点、改善预后等提供了有价值研究线索和理论基础。本文虽为单中心临床资料，但有一定的样本量，且纳入标准严格，分组明确，随访和数据收集完整，具有较强的说服力。慢性阻塞性肺疾病对非小细胞肺癌术后复发风险存的在不容忽视，值得业内高度关注，更期待系统、多中心深入研究。
